# Comparing two admission respiratory virus testing strategies during periods of increased SARS-CoV-2 incidence: a multicenter cross-sectional study

**DOI:** 10.1017/ash.2025.10163

**Published:** 2025-10-03

**Authors:** Paige Reason, Amanda Stagg, Victoria Williams, Robert Kozak, Xena Li, Lorraine Maze dit Mieusement, Heather Candon, Jeff E. Powis, Jerome A. Leis

**Affiliations:** 1 Sunnybrook Health Sciences Centre, Toronto, ON, Canada; 2 Michael Garron Hospital, Toronto, ON, Canada; 3 Shared Hospital Laboratory, Toronto, ON, Canada; 4 Centre for Quality Improvement and Patient Safety, University of Toronto, Toronto, ON, Canada; 5 Temerty Faculty of Medicine, University of Toronto, Toronto, ON, Canada

## Abstract

In this multicenter cross-sectional study of two different approaches to universal SARS-CoV-2 admission testing, hybrid testing (SARS-CoV-2 for asymptomatic and multiplex for symptomatic) resulted in increased isolation days due to ordering errors among symptomatic patients. Universal multiplex testing minimized such errors while detection of asymptomatic non-SARS-CoV-2 viruses remained infrequent.

## Introduction

Syndromic multiplex testing is the foundation of hospital surveillance for seasonal respiratory viruses and multiple international societies have revised recommendations to no longer routinely perform asymptomatic SARS-CoV-2 testing on admission.^1,2^ Despite this, targeted asymptomatic admission testing for SARS-CoV-2 may be beneficial during periods of increased community incidence especially on units with multi-bed rooms or wandering patients.^3,4^

In these situations, implementation may involve a hybrid testing approach (ie, multiplex respiratory virus testing for symptomatic and SARS-CoV-2 testing for asymptomatic) or universal multiplex respiratory virus testing (ie, inclusive of SARS-CoV-2) for all patients on admission. The former approach offers more precise management at the risk of the wrong test being ordered for symptomatic patients. The latter approach may minimize clinician ordering errors at increased cost (eg, additional PCR reagents and technologist time) and risk of detecting asymptomatic non-SARS-CoV-2 respiratory viruses of unclear significance.

We performed the following multicenter cross-sectional study to formally compare these approaches to implementation of universal SARS-CoV-2 testing. Our objective was to determine which of the two implementation approaches resulted in fewer additional isolation days—whether from the wrong test being ordered or from detection of asymptomatic non-SARS-CoV-2 respiratory viruses.

## Methods

We performed a multicenter cross-sectional study comparing two implementation approaches to universal SARS-CoV-2 testing across two acute care hospitals in Toronto, Canada. Both hospitals have a large proportion of shared rooms and maintained admission testing during peak respiratory virus season from December 1^st^ to March 31^st^, during two consecutive years, 2022/23 and 2023/24.

Hospital A implemented hybrid testing while Hospital B implemented expanded multiplex respiratory virus testing. Admission viral testing was conducted at both hospitals via nasopharyngeal or midturbinate swabs within 72 hours of admission and processed at the same laboratory ($38 CAD for SARS-CoV-2 and $59 CAD for multiplex testing). The multiplex RT-PCR panel included targets for SARS-CoV-2, influenza A and B, respiratory syncytial virus, human metapneumovirus, adenovirus, parainfluenza types 1–4, human coronaviruses (OC43, 229E, HKU1, NL63), and rhinovirus/enteroviruses.^5^ Turn-Around-Time (TAT) was approximately 24 h. Reasons for syndromic testing were similar across both hospitals including for acute respiratory symptoms, fever, sore throat, conjunctivitis, myalgia, or other unexplained acute change in clinical status such as a fall or confusion in a patient over 80 years of age. All patients with asymptomatic viral infection were prospectively observed for development of symptoms within 24 h and roommate contacts were prospectively followed until 5 days and tested upon development of symptoms. Transmission-based precautions at both hospitals were used for symptomatic patients and asymptomatic SARS-CoV-2 only. These precautions included private room, use of N95 or medical mask, eye protection, gown, and gloves. Precautions were not routinely initiated for non-SARS-CoV-2 asymptomatic patients but sometimes occurred pending assessment by Infection Prevention & Control.

The primary outcome was the number of additional isolation days compared across both hospitals, defined as additional patient days in transmission-based precautions due to either the wrong test ordered or detection of asymptomatic non-SARS-CoV-2 virus. The number of additional isolation days was expressed as a proportion of overall hospital isolation days, to account for differing facility size. Total isolation days included all patients inclusive of laboratory-confirmed and test-negative patients. Ordering errors were defined as the proportion of patients who underwent SARS-CoV-2 testing who had multiplex testing within 24 h due to being symptomatic.

The fidelity of universal SARS-CoV-2 testing, and proportion undergoing SARS-CoV-2 versus multiplex testing was also compared along with the rate of ordering errors among symptomatic patients, the detection rate of asymptomatic non-SARS-CoV-2 viruses per admission, and the secondary attack rate of asymptomatic non-SARS-CoV-2 cases within shared rooms. Differences in proportions across hospitals were compared using the χ^2^ test. Research ethics review was not required because the project met criteria for exemption based on institutional process for quality improvement at both institutions.

## Results

Adherence to admission screening as a proportion of all admissions was higher in Hospital B compared to Hospital A (6893/7939, 86.8% vs 11 690/14586, 80.1%; *P* < .0001). Use of SARS-CoV-2 testing was significantly higher in Hospital A (40.2% vs 4.2%; *P* < .0001) while multiplex testing was much higher in Hospital B (82.6% vs 39.9%; *P* < .0001).

Table 1 summarizes process and balancing measures per admission. Order errors resulting in repeat testing in symptomatic patients was higher in Hospital A (419/5864, 7.2%) and remained zero at Hospital B (0/334, 0%; *P* < .0001). In contrast, Hospital B incurred higher rates of detection of asymptomatic non-SARS-CoV-2 virus (59/7939, 0.74%) compared with Hospital A (76/14586, 0.52%; *P* = .04). Among detected asymptomatic non-SARS-CoV-2 cases across both sites, 3(2.4%) developed symptoms within 24 h. Secondary attack rate of asymptomatic non-SARS-CoV-2 virus was zero among 57 roommate contacts including 8 cases of asymptomatic influenza.

**Table 1.** Differences in key process and balancing measures between Hospital A with hybrid testing strategy (symptomatic multiplex, asymptomatic SARS-CoV-2 only) and Hospital B with expanded multiplex for all inpatient admissions

	2022/23		2023/24		Combined		
	Hospital A	Hospital B	Hospital A	Hospital B	Hospital A	Hospital B	*p*-value
Proportion of admissions undergoing multiplex testing (%)	2868/7050 (40.7)	2900/4168 (69.6)	2958/7536 (39.3)	3659/3771 (97.0)	5826/14586 (39.9)	6559/7939 (82.6)	< .0001
Proportion of admissions undergoing SARS-CoV-2 testing (%)	2791/7050 (39.6)	319/4168 (7.7)	3073/7536 (40.8)	15/3771 (0.4)	5864/14586 (40.2)	334/7939 (4.2)	< .0001
Ordering errors among symptomatic patients (%)^	270/2791 (9.7)	0/319 (0)	114/3073(3.7)	0/15(0)	419/5 864 (7.2)	0/334 (0)	< .0001
Asymptomatic respiratory viruses detected per all admission (%)	36/7050 (0.5)	25/4168 (0.6)	40/7536 (0.5)	34/3771 (0.9)	76/14586 (0.5)	59/7939 (0.7)	.04

^Proportion of patients tested for SARS-CoV-2 that required multiplex due to symptoms.

There were a total of 5281 isolation days at Hospital A and 4431 isolation days at Hospital B. Table 2 shows the net impact of both testing strategies on isolation days. Both the absolute number and proportion of additional isolation days (419 vs 0, 7.9% vs 0%) due to the wrong diagnostic specimen ordered outweighed the isolation days from asymptomatic non-SARS-CoV-2 detection (10 vs 17, 0.2% vs 0.4%).

**Table 2.** Net impact of two admission respiratory virus testing strategies on additional isolation days

Cause of additional isolation days	Hospital A, total additional isolation days—n(% of all isolation days)	Hospital B, total additional isolation days—n (% of all isolation days)	Net difference
Wrong diagnostic test ordered (SARS-CoV-2 ordered in symptomatic patient)	419 (7.9)	0 (0)	419 (7.9)
Detection of non-SARS-CoV-2 respiratory viruses	10 (0.2)	17 (0.4)	7 (0.2)

## Discussion

This multicenter cross-sectional study can help inform the approach to implementing universal respiratory virus admission testing should the need arise. A hybrid testing approach resulted in increased ordering errors among symptomatic patients resulting in delays in discontinuation of precautions while awaiting results of appropriate multiplex testing. The universal multiplex approach avoided these ordering errors and despite slightly higher rate of detection of asymptomatic non-SARS-CoV-2 viruses, was associated with fewer additional isolation days.

While the role of asymptomatic admission testing for SARS-CoV-2 is limited to exceptional circumstances, its role for other respiratory viruses is even less clear. Some evidence suggests that influenza can be transmitted without symptoms.^6^ Our study did not identify transmission among asymptomatic influenza cases detected but the number remained small. Across many jurisdictions in the United States and Canada, multiplex respiratory panels may not be government-funded and a major barrier to its use for universal admission testing. Given lack of demonstrated benefit in preventing non-SARS-CoV-2 infection, the main benefit of universal multiplex testing was the simplicity of implementation which eliminated errors.

If a hybrid testing approach is used for universal admission testing, ordering errors are an important quality assurance indicator to prevent delays in discontinuation of precautions. In our study, these errors were highest during the first season following implementation and decreased during season two likely due to improved training and education. This finding highlights the importance of staff training if using a hybrid testing approach to reduce the unwanted outcome of unnecessary isolation days. The challenge is that hybrid testing may only be beneficial during short periods of high SARS-CoV-2 incidence and therefore difficult to implement reliably during such compressed timeframes.

This study has several limitations. First, it is an observational study with potential for confounding that could have affected respiratory virus testing between hospitals. However, both hospitals are similarly situated in proximity where community rates would have been similar. Second, uptake of either testing strategy could vary based on contextual factors including healthcare worker training and adherence to Infection Prevention measures. Approximately 20% of admissions across the two hospitals missed admission screening likely due to a delay in specimen collection and/or a short admission with discharge prior to testing.

Neither hospital continues asymptomatic admission testing at this time in accordance with updated local public health guidelines.^7^ Should the need arise in the future, adding SARS-CoV-2 testing for asymptomatic patients must actively address ordering errors among symptomatic patients to minimize increase in isolation days. Having a single multiplex test available for universal use carries some additional cost but may facilitate implementation and avoid these unintended consequences.
